# KLK6 proteolysis is implicated in the turnover and uptake of extracellular alpha-synuclein species

**DOI:** 10.18632/oncotarget.13264

**Published:** 2016-11-10

**Authors:** Georgios Pampalakis, Vasia-Samantha Sykioti, Methodios Ximerakis, Ioanna Stefanakou-Kalakou, Ronald Melki, Kostas Vekrellis, Georgia Sotiropoulou

**Affiliations:** ^1^ Department of Pharmacy, School of Health Sciences, University of Patras, Rion-Patras, Athens, Greece; ^2^ Center for Neurosciences, Biomedical Research Foundation, Academy of Athens, Athens, Greece; ^3^ Paris-Saclay Institute of Neuroscience, Centre National de la Recherche Scientifique, Gif-Sur-Yvette, France

**Keywords:** KLK6, α-synuclein, degradomics, metalloproteases, in vitro substrates

## Abstract

KLK6 is a serine protease highly expressed in the nervous system. In synucleinopathies, including Parkinson disease, the levels of KLK6 inversely correlate with α-synuclein in CSF. Recently, we suggested that recombinant KLK6 mediates the degradation of extracellular α-synuclein directly and *via* a proteolytic cascade that involves unidentified metalloproteinase(s). Here, we show that recombinant and naturally secreted KLK6 can readily cleave α-synuclein fibrils that have the potential for cell-to-cell propagation in “a prion-like mechanism”. Importantly, KLK6-deficient primary cortical neurons have increased ability for α-synuclein fibril uptake. We also demonstrate that KLK6 activates proMMP2, which in turn can cleave α-synuclein. The repertoire of proteases activated by KLK6 in a neuronal environment was analyzed by degradomic profiling, which also identified ADAMTS19 and showed that KLK6 has a limited number of substrates indicating specific biological functions such as the regulation of α-synuclein turnover. We generated adenoviral vectors for KLK6 delivery and demonstrated that the levels of extracellular α-synuclein can be reduced by neuronally secreted KLK6. Our findings open the possibility to exploit KLK6 as a novel therapeutic target for Parkinson disease and other synucleinopathies.

## INTRODUCTION

The link between α-synuclein and Parkinson's disease (PD) and other neurodegenerative diseases collectively called synucleinopathies is well documented [1]. Although α-synuclein lacks a signal peptide, it is found in secretory vesicles and it is secreted into the extracellular milieu of α-synuclein-overexpressing cells and, importantly, in the plasma and cerebrospinal fluid (CSF) of healthy humans and PD patients [2]. Emerging evidence suggests that extracellular α-synuclein may be implicated in the pathology of PD. According to a “prion-like” hypothesis, α-synuclein fibrils but not monomers can spread from one cell to another, to seed the intracellular α-synuclein monomers in a manner that resembles prion diseases, thus, accelerating PD pathology [3, 4]. It is plausible that deregulation in the normal processing of secreted α-synuclein contributes to the formation of “toxic” α-synuclein species and fibrils and as such it may be a causative risk factor for PD. In this respect, proteolytic processing of extracellular α-synuclein emerges as a new important field for active investigation with potential implications for therapy.

Of the human proteases, MMPs, plasmin, and KLK6 have been shown to cleave α-synuclein *in vitro*. Limited proteolysis of α-synuclein by MMP3 promotes its aggregation [5] and the resulting aggregates exhibit enhanced cytotoxicity [6]. In the order of decreased efficiency, MMP14, 2, 1 and 9 can also cleave α-synuclein [6]. MMP1 promotes α-synuclein aggregation but MMP9 does not alter it [5]. Finally, it was reported that PD patients have reduced levels of MMP2 in substantia nigra [7]. Plasmin is also able to cleave and degrade the extracellular α-synuclein [8]. Contrary to MMP3 that cleaves at the C-terminus [5, 6], plasmin mainly cleaves at the N-terminus. C-terminally truncated α-synuclein has a greater tendency for aggregation [9, 10] and approximately 15% of α-synuclein in Lewy bodies is C-terminally truncated [11]. Cleavage of α-synuclein by KLK6 yielded proteolytic fragments that inhibited the aggregation of the intracellular α-synuclein, as shown by Iwata et al. [12], who claimed that KLK6 is localized in mitochondria and, upon stress it is released into the cytoplasm to cleave α-synuclein. However, a later study failed to detect KLK6 in the mitochondria nonetheless, they showed that endogenously secreted KLK6 could cleave the extracellular α-synuclein [13]. Whether KLK6 can cleave both the wt and mutant forms of α-synuclein with a similar efficiency is currently in controversy. Iwata et al. [12] found that recombinant A53T α-synuclein was resistant to KLK6 cleavage but Kasai et al. [14] claimed that both recombinant wt and A53T forms could be cleaved with the same efficiency.

Human KLK6 was originally identified by differential display for its potential implication in breast and ovarian cancer and named protease M [15]. A year later, myelencephalon-specific protease (MSP), the rat homologue of KLK6 was cloned, and shown to be induced in the rat spinal cord after excitotoxic injury [16]. In humans, KLK6 is expressed at high levels in the nervous system and is one of the few most abundant serine proteases in the CSF where it is secreted at mg/l [17–19]. CSF levels of KLK6 are reduced in patients with synucleinopathy including PD [20–22]. Previously, we showed that KLK6 is able to cleave the recombinant α-synuclein (wt and A53T mutant) efficiently. However, the endogenously secreted α-synuclein was resistant to KLK6 cleavage but became proteolysis-susceptible upon delipidation. Our experiments indicated that KLK6 could activate as yet unidentified downstream metalloprotease(s) to cleave the naturally secreted α-synuclein in a cascade manner [23].

Here, we identified MMP2 as the elusive protease activated by KLK6 to cleave the α-synuclein. N-terminomic mapping of global proteolytic cleavage events putatively catalyzed by KLK6 identified that ADAMTS19 metalloprotease is also activated by KLK6 and may participate in proteolytic pathways leading to α-synuclein processing. In line with the state-of-the art “prion-like” mechanism of α-synuclein spreading of PD pathology, we tested whether α-synuclein fibrils that have the ability to propagate and spread from cell-to-cell could be proteolyzed by KLK6. We show that KLK6 (both the recombinant and naturally secreted) can cleave the fibrilar α-synuclein. This provides the first time evidence that KLK6 could be a major player in the turnover of α-synuclein species.

## RESULTS

### Proteolytic processing of α-synuclein entails a metalloprotease, which must be activated by KLK6

**Figure 1:**
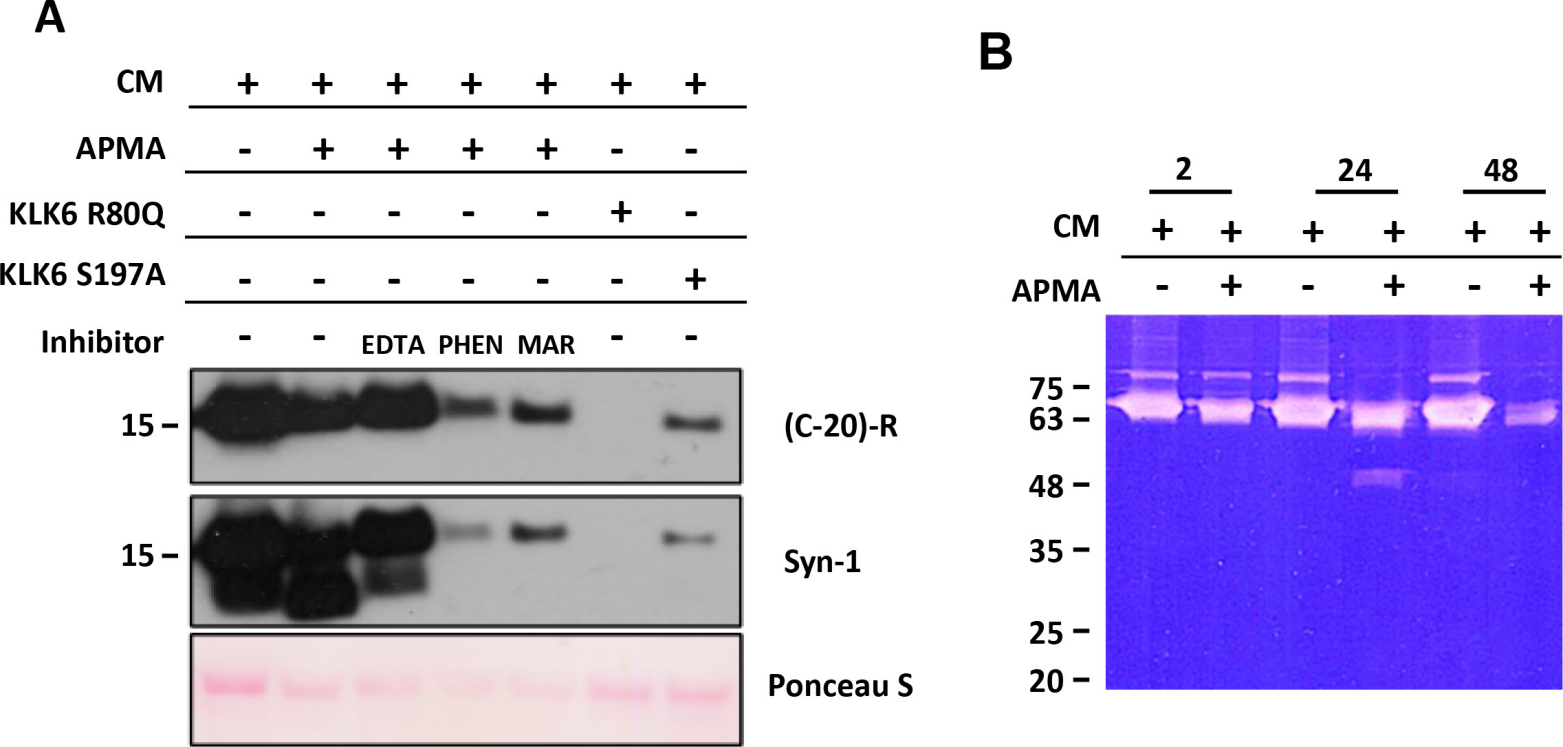
A metalloproteinase mediates α-synuclein degradation (**A**) APMA addition to SH-SY5Y cell supernatant results in cleavage of secreted α-synuclein. Cleavage is inhibited by 10 mM EDTA or 10 mM 1,10-phenanthroline (PHEN) or 10 μM marimastat (MAR). Recombinant KLK6 R80Q (50 nM) cleaves the endogenous α-synuclein, as shown previously [23]. (**B**) ProMMP activation by APMA in SH-SY5Y CM monitored with gelatin zymography. Activation of the metalloprotease takes place after 24 hours, while after 48 hours degradation and reduction in proteolytic activities occurs. Molecular sizes are shown in kDa. APMA, 4-aminophenylmercury acetate; CM, conditioned media. (C-20)-R, antibody recognizing the C-terminus of α-synuclein. KLK6 R80Q corresponds to the stabilized KLK6 enzyme that resist autodeactivation by cleavage at arginine 80 (R80) [29], while the KLK6 S197A is the inactive KLK6 enzyme due to mutation at the active site Ser. The experiments have been carried out twice.

To investigate the involvement of metalloproteases in the cleavage of α-synuclein, we added 4-aminophenylmercury acetate (APMA), a general metalloprotease activator [24], into the secretome of SH-SY5Y cells that physiologically secrete α-synuclein [25]. Figure 1A shows that cleavage of endogenous α-synuclein can occur, upon APMA, in the absence of KLK6. The α-synuclein cleavage was fully inhibited by EDTA and 1,10-phenanthroline general metalloprotease inhibitors but also by marimastat [26], a more selective MMP inhibitor (Figure 1A). Taken together, these results confirm that metalloprotease activity is involved in the proteolysis of α-synuclein. RT-PCR and Western blotting verified that KLK6 is not expressed by naïve or differentiated SH-SY5Y cells (data not shown). SH-SY5Y secretomes in which APMA was added, showed a gelatinolytic activity of approximately 65–70 kDa (Figure 1B). Based on the size and its inhibition by marimastat, we hypothesized that it corresponds to active MMP2, and that this is likely the protease activated by KLK6. This is corroborated by the generation of the 45 kDa gelatinolytic band corresponding to active MMP2. It is known that active MMP2 of 65–70 kDa is autocatalytically cleaved at the C-terminus yielding a truncated active form of 45 kDa that lacks the C-terminal domain; this is deactivated by degradation at 48 hours [27].

### KLK6 activates proMMP2

**Figure 2:**
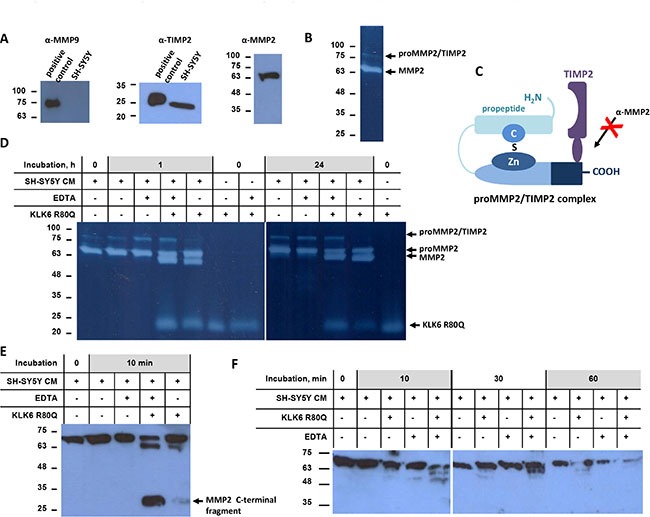
KLK6 activates proMMP2 (**A**) Western blot analysis of SH-SY5Y cell supernatants for detection of MMP9, TIMP2, and MMP2. Positive controls for MMP9 included U2OS cells stimulated with TPA and for TIMP2 MDA-MB-231 cells. (**B**) Gelatin zymography showed two bands that correspond, to proMMP2 and potentially to proMMP2/TIMP2, respectively. (**C**) Schematic diagram of the proMMP2/TIMP2 complex showing coverage of the C-terminal domain of proMMP2 that is recognized by the anti-MMP2 antibody that could account for the band detected around 100 kDa. (**D**) Time course of proMMP2 activation in SH-SY5Y cell supernatant. EDTA did not inhibit proMMP2 activation. Proteolytic activities were detected by gelatin zymography. (**E**) Activation of proMMP2 monitored by Western blotting with a specific antibody for the C-terminus of proMMP2. (**F**) Time-course of proMMP2 activation indicated by the gradual decrease of the proMMP2 form. The first antibody used binds specifically the propeptide of proMMP2, which is removed upon activation. The experiments were carried out three times.

Gelatin zymography and Western blotting showed that SH-SY5Y secrete proMMP2 and TIMP2 but not proMMP9 (Figure 2A). Two bands were detected, a strong band around 70 kDa that likely corresponds to proMMP2 and a band of about 100 kDa that could be a SDS-resistant complex of a metalloprotease with inhibitor [28] (Figure 2B), as for example with TIMP2 (Figure 2C). Addition of exogenous KLK6 in SH-SY5Y CM resulted in a time-dependent activation of proMMP2, as indicated by the generation of the 64 kDa band corresponding to active MMP2, while the ~20–25 kDa is due to the gelatinolytic activity of KLK6 (Figure 2D). Activation of proMMP2 is shown by reduction of its size upon removal of the propeptide (Figure 2E). Activation of proMMP2 is further shown by the appearance of the 30–35 kDa band that corresponds to autocatalytically truncated MMP2 at the C-terminus. EDTA enhanced proMMP2 activation by KLK6. This could be attributed to a conformational change of proMMP2 induced by removal of Ca^2+^ rendering it more susceptible to cleavage by KLK6. Figure 2F shows the time-course of proMMP2 activation by KLK6 using an antibody that recognizes the propeptide (activation peptide). The gradual decline and eventual disappearance of the 72 kDa band further proves that proMMP2 is indeed activated by KLK6.

**Figure 3:**
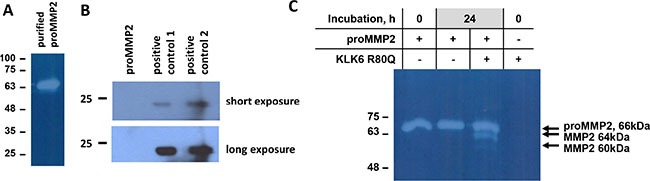
Activation of purified proMMP2 by KLK6 (**A**) Gelatin zymogram of purified proMMP2 by a two-step affinity chromatography protocol. (**B**) TIMP2 is not present in the purified proMMP2 preparation, as shown by Western blotting. Positive controls 1 and 2 are supernatants from MDA-MB-231 and T47D cells, respectively. (**C**) Activation of purified proMMP2 by KLK6 shows the same pattern as in SH-SY5Y supernatants. The experiments were carried out three times.

The ability of KLK6 to directly activate the proMMP2 was additionally verified with purified proteins. Indeed, the MMP2 pro-peptide contains multiple Lys and less Arg residues (Uniprot: P08253). Endogenous proMMP2 was purified from SH-SY5Y CM by two-step affinity chromatography (Figure 3A). TIMP2 was not detected in the proMMP2 preparation (Figure 3B). Incubation of the purified proMMP2 with KLK6 resulted in its activation (Figure 3C). The gelatinolytic pattern of purified proMMP2 activation was similar to that observed in SH-SY5Y CM which proves that no other protease is involved in proMMP2 activation. Finally, Supplementary Figure S1 shows that KLK6 is not cleaved by the purified proMMP2 activated by APMA and that KLK6 is stable in SH-SY5Y CM. Proteomic profiling of the SH-SY5Y secretome verified that indeed MMP2 is present. MMP9 or other MMPs were not detected except for the ADAMTS 4, 7, 9, 1, 19 (data not shown).

### Identification of KLK6 cleavage sites by TAILS

**Figure 4:**
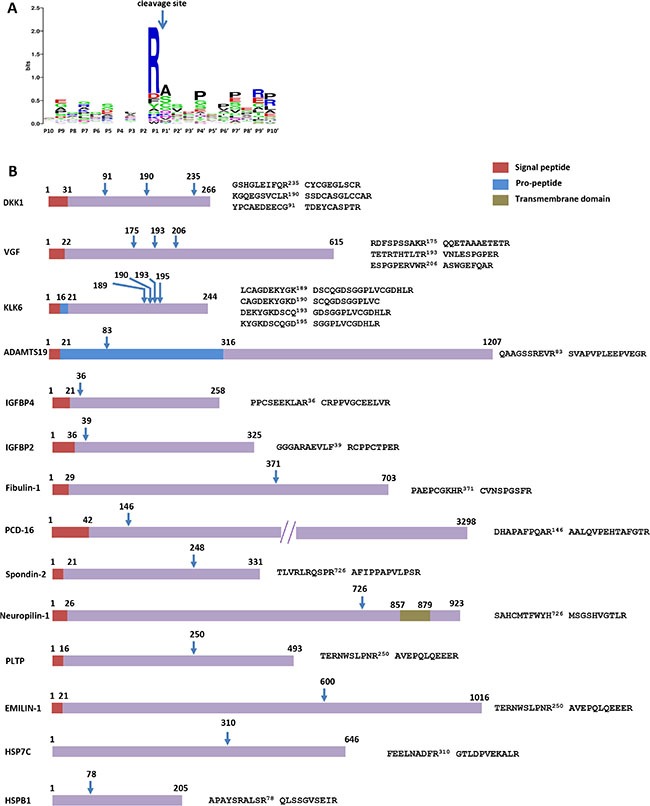
Degradomic repertoire of KLK6 treated secretome (**A**) Sequence logos generated with WebLogo based on the identified proteolytic events show a preference for cleavage after Arg residues. (**B**) Schematic diagrams of the cleavage events and respective cleavage sites on protein substrates. Only cleavages found in both experiments with Fc > 1 are shown. DKK1: Dickkopf-related protein 1; VGF: Neurosecretory protein VGF; ADAMTS19: A disintegrin and metalloproteinase with thrombospondin motifs 19; IGFBP4 or 2: Insulin-like growth factor-binding protein 4 or 2; PCD-16: Protocadherin-16; PLTP: Phospholipid transfer protein; HSP7C: Heat shock cognate 71 kDa protein; HSPB1: Heat shock protein beta-1.

To identify the protein repertoire that is cleaved by KLK6 in the SH-SY5Y secretome, TAILS of supernatants was performed under conditions of < 6% cell death (data not shown). SH-SY5Y CM were incubated with 50 nM recombinant active KLK6 R80Q or inactive KLK6 S197A for 24 hours. TAILS identified and quantified the N-termini generated after KLK6 cleavage in SH-SY5Y CM. The results shown in Supplementary Tables S1 and S2 are from two independent experiments in which we compared the N-termini in the secretomes containing the active *vs* the inactive KLK6 (as negative control). Initially, we focused on the 192 N-termini of putatively secreted or membrane associated proteins that were present in both experiments. Filtering the list for cleavage events that are more than two-fold enriched in both experiments upon incubation with active KLK6 highlighted 38 proteolytically generated N-termini from 31 different protein substrates (Supplementary Table S3 and Figure 4A and 4B). KLK6 has an annotated preference for Arg > Lys at the P1 position [29].

Among the putative KLK6 substrates that were found in both replicates, we noticed components of BMP/Wnt and IGFBPs signaling (Table 1). In addition, KLK6 can cleave the latent transforming growth factor binding proteins 3 (LTBP3) and 4 (LTBP4) at Arg residues (Supplementary Figure S2). Consequently, KLK6 may be implicated in the activation of the TGF-β pathway. Likewise, a link between KLK proteases and TGF-β signaling as well as IGFBPs has been previously suggested [30]. Furthermore, we notice that in both replicates, KLK6 cleaves ADAMTS19 post-arginine at position 83, thus removing the pro-domain. This finding is suggestive of KLK6 being able to activate pro-ADAMTS19.

**Table 1:** Pathways regulated by KLK6 proteolysis

Pathway	Protein	TAILS	Position
WNT/BMP	Dickkopf-related protein 1; DKK1	6.741,7.6543.041, 4.533.0412.221	R^235^ R^190^ T^180^ G^91^
	Na(+)/H(+) exchange regulatory cofactor NHE-RF1; SLC9A3R1	6.741,3.2141.25	R^179^ R^219^
IGFs	Insulin-like growth factor-binding protein 2; IBP2	5.7415.741, 5.7024.42	K^215^ F^39^ E^192^
	Insulin-like growth factor-binding protein 4; IBP4	5.157, 4.4603.741	R^36^ R^48^
	Insulin-like growth factor 2; IGF2	9.9664.1561.315, 1.978	E^35^ G^64^ R^63^
TGFs	Latent-transforming growth factor beta-binding protein 4; LTBP4	9.9669.9662.934	R^622^ R^472^R ^335^
	Latent-transforming growth factor beta-binding protein 3; LTBP3	9.9663.157	R^377^ F^724^

MMP2 has not been detected by this analysis probably due to low abundancy. It should be reminded that α-synuclein is fully degraded by KLK6, thus it could not be detected by TAILS that can only identify the newly formed N-terminal sequences yielded by specific cleavage events (limited proteolysis).

### Cleavage of naturally secreted α-synuclein by cell-secreted KLK6

**Figure 5:**
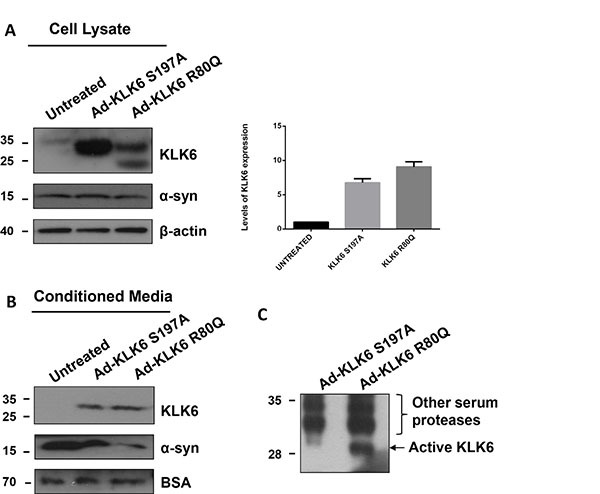
KLK6 is constitutively secreted following its overexpression in primary cortical neurons and reduces secreted α-synuclein protein levels Adenoviral vectors that drive the expression of stabilized active KLK6 R80Q (Ad-KLK6 R80Q) or inactive KLK6 S197A (Ad-KLK6 S197A) were generated and used to transduce (50 multiplicity of infection) primary neuronal cortical cultures prepared from wt mice (embryonic day 16). 72 hours post-infection cells and CM were collected. (**A**) Cell lysates (40 μg) and (**B**) CM concentrated 50-fold were subjected to electrophoresis and analyzed by immunoblotting, using the syn-1 and the KLK6 antibodies. β-actin and BSA were used as loading controls. Protein levels of virally expressed active (R80Q) and inactive (S197A) KLK6 in cell lysates were evaluated by densitometry quantification. Band intensities were normalized versus β-actin. Bars represent the mean ± S.D. of three independent experiments. (**C**) Detection of active KLK6 using an activity-based probe (ABP) developed in-house (our unpublished data). The ABP carries an organophosphorus ester as a reactive group and a biotin tag. β-actin and BSA were used as loading controls.

To evaluate whether cell-secreted KLK6 could cleave secreted α-synuclein, we transduced five day-old primary cortical neurons with adenoviruses driving the expression of constitutively active KLK6 R80Q and inactive KLK6 S197A, using 50 multiplicities of infection (MOIs) for 24 hours. Initially, we tested the expression of KLK6 (R80Q and S197A) 72 hours post-infection by immunoblotting. As shown in Figure 5A, both forms of KLK6 are highly expressed. Densitometric analysis revealed a 5- and 9-fold increase of adenovirally expressed KLK6 active and inactive forms, respectively, when compared to endogenous levels. As expected, KLK6 is also secreted in the extracellular space and detected in abundant levels in the CM (Figure 5B). We verified that the adenovirally expressed KLK6 R80Q was active in our CM (Figure 5C) using an activity-based probe developed *in house* (our unpublished data). Subsequently, we assessed the impact of KLK6 over-expression on α-synuclein levels using an antibody against α-synuclein. Interestingly, intracellular levels of α-synuclein were not affected by intracellular KLK6 expression; however, adenoviral expression of active KLK6 R80Q protein in the CM led to the degradation of extracellular α-synuclein. Supplementary Figure S3 demonstrates that adenoviral infection does not affect the survival of primary neuronal cortical cultures.

### KLK6 cleaves α-synuclein fibrilar forms

**Figure 6:**
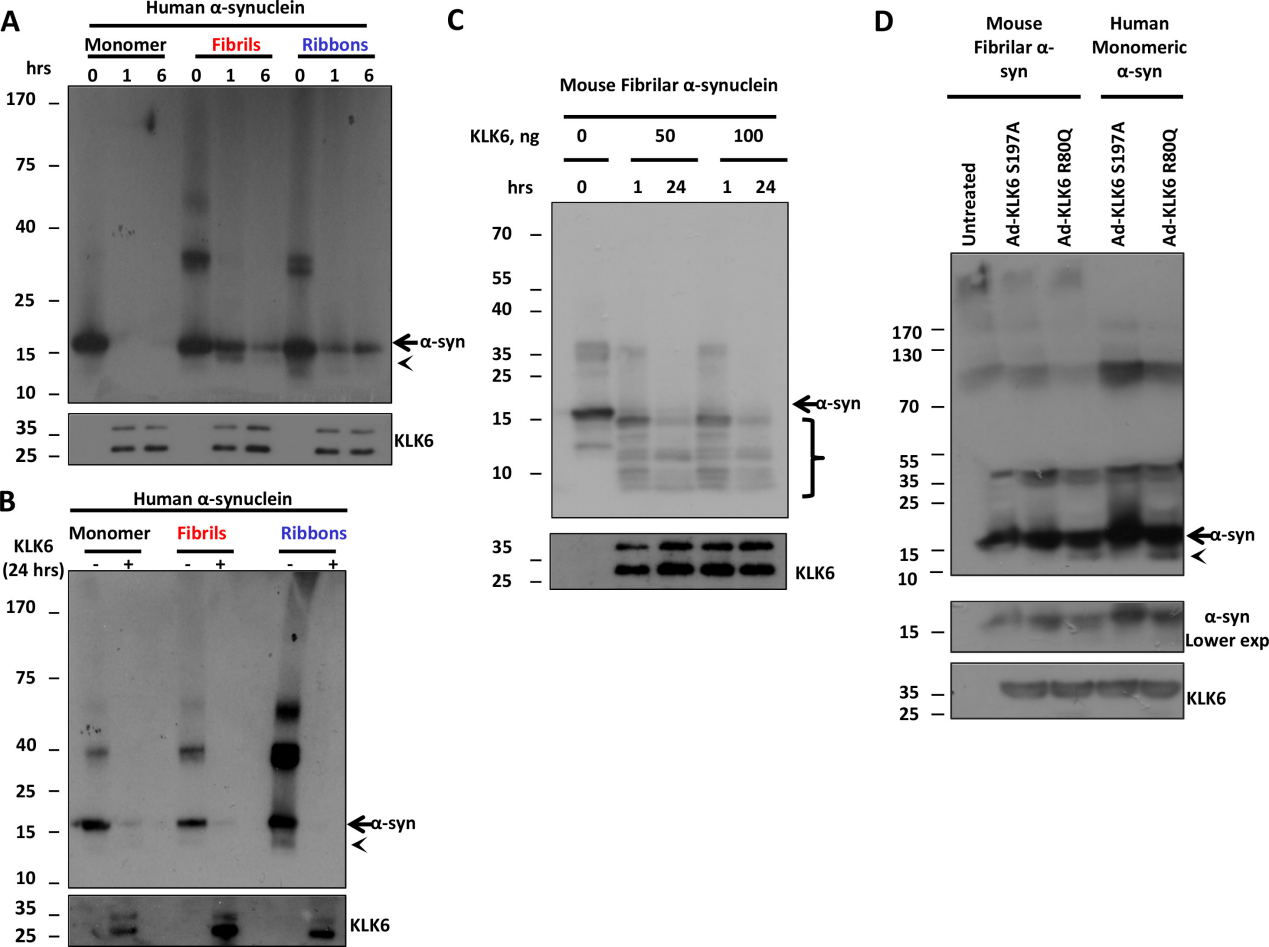
KLK6 readily cleaves fibrilar α-synuclein species (**A**) 250 ng of human recombinant α-synuclein (monomer, fibrils and ribbons) were incubated with 19 ng of the recombinant active (R80Q) form of KLK6 for 0, 1 and 6 hours at 37°C. Samples were subjected to electrophoresis and α-synuclein degradation was examined by immunoblotting using the syn-1 antibody. (**B**) The same reaction as in A was performed for 24 hours and samples were analyzed as above. (**C**) 250 ng of mouse recombinant fibrilar α-synuclein was incubated with 50 ng and 100 ng of the recombinant active (R80Q) form of KLK6 for 1 and 24 hours at 37°C. Samples were analyzed as described in (A). (**D**) Adenoviral vectors (50 MOI) that drive the expression of constitutively active KLK6 R80Q or inactive KLK6 S197A were used to transduce primary neuronal cortical cultures prepared from wt mice. 72 hours post-infection, CM were collected, concentrated (50-fold concentration) and incubated with 250 ng of mouse recombinant fibrilar α-synuclein for 24 hours at 37°C. The presence of α-synuclein proteolytic fragments was assessed by immunoblotting, using the (C-20)-R antibody. The presence of KLK6 in the reactions was verified by using the KLK6 antibody. Arrows indicate full size α-synuclein, arrowheads and brackets indicate α-synuclein proteolytic fragments.

Since fibrilar forms of α-synuclein are considered neurotoxic, we sought to investigate whether KLK6 has the capacity to proteolytically process toxic α-synuclein species. Two different pure fibrilar strains of α-synuclein known as fibrils and ribbons based on their structure under transmission electron microscopy were used. Both strains have been shown to exhibit enhanced propagation capacity and toxicity [31]. Fibrils are toxic strains that result in progressive motor impairment *in vivo*, while ribbons cause histopathological phenotype that resembles PD and multiple system atrophy [32]. We incubated these forms of α-synuclein with the recombinant active KLK6 R80Q and samples were analyzed by immunoblotting. As shown in Figure 6A and 6B enzymatically active KLK6 R80Q is able to cleave human recombinant α-synuclein fibrils and ribbons in a time and dose-dependent manner. Further, we examined whether the recombinant active KLK6 R80Q could cleave mouse pre-formed fibrilar (PFF) α-synuclein that has been shown to significantly accelerate the propagation and seeding of the endogenous α-synuclein *in vivo* [4]. Incubation of PFFs with KLK6 for 1 and 24 hours resulted in a marked decrease of α-synuclein levels and the appearance of proteolytic fragments as early as 1 hour (Figure 6C). Collectively, these data indicate that KLK6 is able to cleave both monomeric and toxic fibrilar forms of α-synuclein and suggest that KLK6 can, thus, potentially affect the seeding and propagation of α-synuclein.

### Cell-secreted KLK6 can cleave α-synuclein fibrils

To examine whether cell-secreted KLK6 is able to cleave pre-formed α-synuclein fibrils (PFF), we transduced 5 day-old primary cortical neurons with adenoviruses expressing the active and inactive form of KLK6. 72 hours post-infection CM were collected, concentrated and incubated with either recombinant human monomeric or mouse pre-formed fibrilar α-synuclein. As depicted in Figure 6D, cell-secreted active KLK6 R80Q readily cleaved human monomeric and mouse fibrilar α-synuclein, leading to the appearance of proteolytic peptide fragments below 15 kDa.

### Absence of KLK6 enhances the internalization and induction of insoluble α-synuclein aggregates in primary neuronal cultures treated with fibrilar α-synuclein species

**Figure 7:**
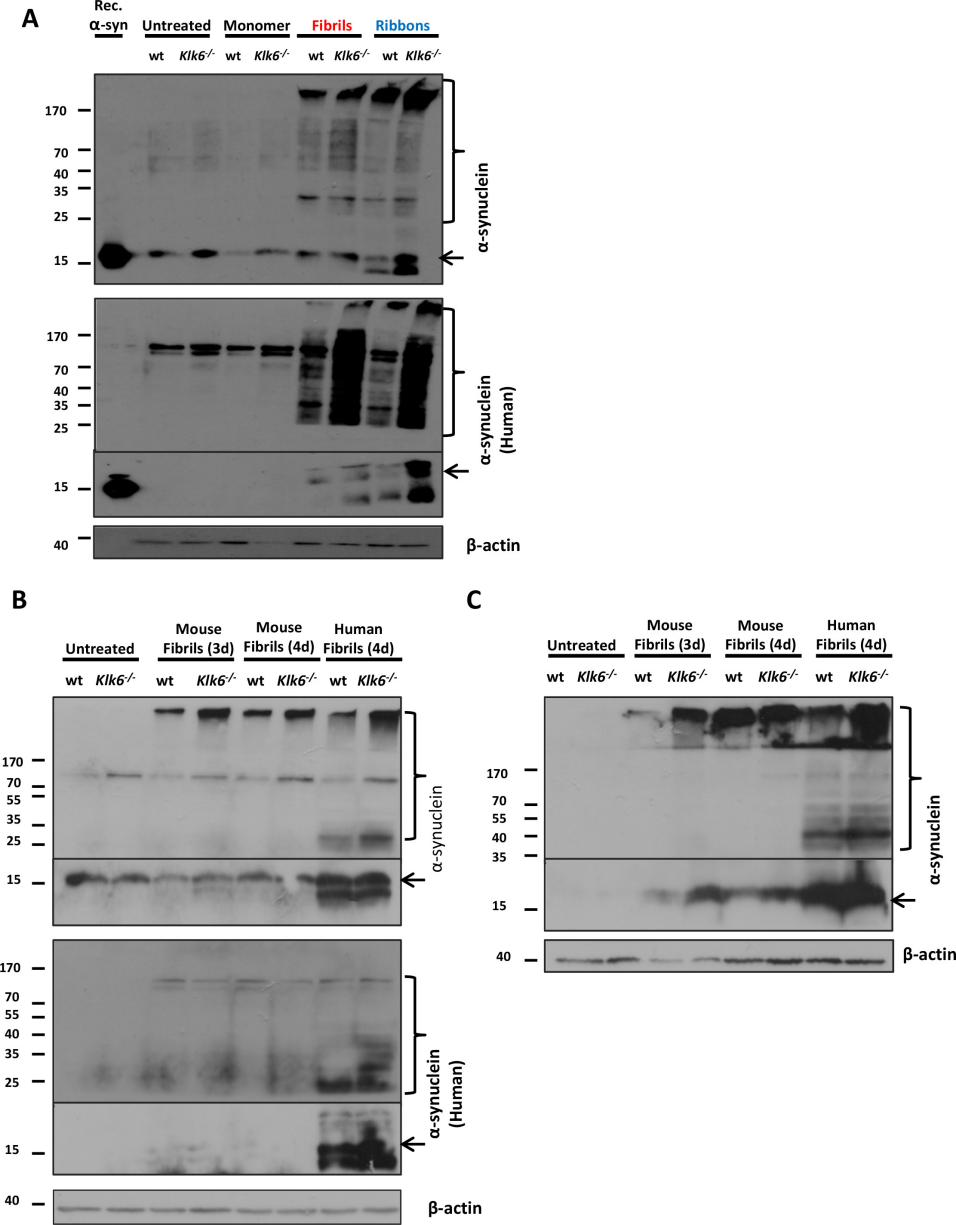
*Klk6^–/–^* primary neuronal cultures internalize exogenously added fibrilar forms of α-synuclein with higher efficiency compared to wt cultures Primary neuronal cortical cultures were prepared from wt or *Klk6^–/–^* mice and were cultivated for 9 days *in vitro*. (**A**) Neuronal cultures were treated with human recombinant forms of α-synuclein (monomer, fibrils and ribbons) for 4 days and (**B**) with mouse or human fibrilar forms of α-synuclein for 3 and 4 days. Neurons were lysed in 1% TX-100 and internalized α-synuclein was detected by immunoblotting, using specific antibodies against either the human-specific (4B12) or total (human and rodent) α-synuclein (C-20)-R. (**C**) To detect insoluble α-synuclein species, lysates were further extracted in 1% sarcosyl and α-synuclein levels were detected using the (C-20)-R antibody. β-actin was used as loading control. Arrows indicate monomeric α-synuclein, brackets indicate α-synuclein higher molecular weight species.

Several studies have reported that exogenous human α-synuclein preformed fibrils, when added to primary neuronal cultures, are internalized and can seed the recruitment of endogenous soluble α-synuclein into insoluble fibrilar aggregates that recapitulate the main features of LBs [33]. The exact mechanism of α-synuclein spreading remains elusive; however extracellular α-synuclein and any imbalances in its clearance mechanisms are considered to be key players of this process and may favor the formation and/or accumulation of oligomeric and fibrilar species that promote the pathology. Since we have shown that recombinant KLK6 is able to readily cleave fibrilar forms of α-synuclein, we sought to investigate in a primary neuronal cellular model whether the absence of this α-synuclein degrading enzyme facilitates the internalization and seeding capacity of exogenously added preformed fibrils. For this reason, we prepared primary neuronal cortical cultures either from wt or *Klk6^–/–^* mice and the neurons were examined 3 or 4 days after the addition of human monomeric α-synuclein, human fibrils or ribbons and mouse fibrilar α-synuclein. The generation and detailed characterization of the *Klk6^–/–^* mice will be the subject of a subsequent paper. Briefly, Supplementary Figure S4 depicts the cassette used for the generation of *Klk6^−/−^* and the genotyping strategy. Immunoblot analyses were conducted on neuronal lysates sequentially extracted with 1% TX-100, followed by 1% sarcosyl. As shown in Figure 7, when probed using antibodies against either the human-specific (4B12) or total (human and rodent) α-synuclein, α-synuclein protein was detected as a main band at ~15 kDa. Accumulation of α-synuclein at higher molecular weights was also observed, indicating the conversion of monomeric α-synuclein into stable oligomeric or fibrilar states. Importantly, *Klk6^–/–^* neuronal cortical cultures seem to internalize fibrilar forms of α-synuclein, both different human fibrilar strains (fibrils and ribbons) and preformed mouse fibrils, with higher efficiency compared to wt neuronal cultures. In contrast to wt primary neuronal cortical cultures, *Klk6^–/–^* treated with fibrilar species show elevated TX-100-soluble α-synuclein levels, accompanied by a concomitant increase of sarcosyl-soluble α-synuclein compared to both non-treated and wt neuronal lysates. These findings indicate that KLK6 seems to be a major degrading enzyme of α-synuclein and that any impairment or dysfunction of this clearing mechanism may be a key contributor to the accumulation, cell-to-cell transmission and propagation of aggregated pathogenic α-synuclein.

## DISCUSSION

Based on the ability of KLK6 to cleave α-synuclein and its decreased expression in patients, KLK6 was linked to PD and other synucleinopathies (reviewed in [34]). Recently, we proposed that KLK6 could degrade the naturally secreted α-synuclein directly and indirectly by involvement of metalloprotease(s) [23]. Here, we show that proMMP2 is activated by KLK6 to cleave the α-synuclein, thus, providing evidence that a proteolytic cascade could be implicated in the turnover of extracellular α-synuclein. Using TAILS, we identified ADAMTS19 as another protease that may be activated by KLK6 and could be part of the cascade. TAILS also revealed that KLK6 recognizes a limited number of protein substrates indicating that KLK6 displays a rather restricted substrate specificity thus it may exert specific regulatory roles that likely include the cleavage of α-synuclein. This is in contrast to previous studies with isolated components claiming KLK6 to be a highly degradative protease [35, 36]. Also, a previous degradomic study of KLK proteases has found a limited number of substrates, thus corroborating selective rather than degradative proteolysis by KLK proteases [30].

**Figure 8:**
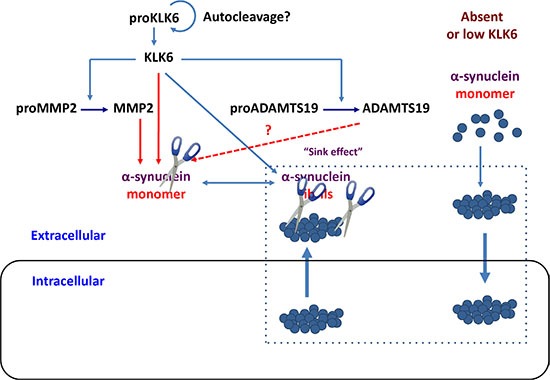
Schematic representation of how KLK6 protease could lead to degradation of intracellular α-synuclein aggregates According to a hypothesis, known as sink effect (*rectangular*), intracellular and extracellular α-synuclein species are in equilibrium, thus, removal of extracellular α-synuclein could result in reduction of its intracellular levels with potential therapeutic applications. We propose that a proteolytic cascade is initiated by KLK6 that activates downstream metalloproteinases, such as MMP2 and ADAMTS19. The output result is the degradation of extracellular α-synuclein monomers and fibrils. Reduction of extracellular α-synuclein levels could lead to clearance of intracellular α-synuclein aggregates. Accumulation of α-synuclein, in the absence of KLK6 proteolysis could promote internalization of fibrilar species. This is supported by results shown in Figure 7, where *Klk6^–/–^* neurons have higher ability to uptake α-synuclein fibrils but not monomers. The α-synuclein monomers (dark blue circles) aggregate to yield fibrilar forms. Scissors depict proteolytic cleavage.

The proteolytic pathways that regulate the levels of α-synuclein are largely unknown. Two intracellular proteases were demonstrated to cleave α-synuclein *in vitro*, namely calpain [37] and cathepsin D [38]. The α-synuclein fragments generated by calpain enhance aggregation indicating that calpain could promote the generation of intracellular aggregates [37]. Neuronal KLK6 protease could be a key enzyme in cleaving secreted α-synuclein [13, 23]. In so doing, KLK6 may also activate other proteases, such as the MMP2 or/and ADAMTS19. Thus, KLK6 could catalyze a cascade “crosstalk” with metalloproteases in the nervous system as shown in Figure 8.

Proteomic profiling identified α-synuclein among the top substrates fully degraded by KLK6 (data not shown), thus, corroborating our hypothesis. For the first time, WNT/BMP, IGF and TGF signaling were identified as pathways activated by KLK6 proteolysis (Table 1). The association of KLK6 with the TGF-β pathway was suggested before [39]. Interestingly, perturbations in TGF-β signaling pathway have been involved in the regulation of survival of dopaminergic neurons and the aggregation of α-synuclein [40].

Further, for the first time here, we provide evidence that KLK6 can degrade fibrilar α-synuclein species that have the ability to propagate and spread PD pathology *in vivo* by a “prion-like” mechanism [31]. Very recently, it was shown that α-synuclein strains can induce MSA in humans. These strains have the ability to propagate and seed the endogenous α-synuclein in mouse brains [41]. That KLK6 can fully degrade different α-synuclein oligomers with a proven propagation capacity indicates that it may represent a novel therapeutic protein for PD therapy. In this direction we generated adenoviral vectors that drive the expression of KLK6 in cortical neurons which resulted in reduced levels of secreted α-synuclein.

Reduction in the levels of extracellular α-synuclein by proteolytic degradation may lower the levels of intracellular α-synuclein (“sink effect”) as depicted in Figure 8. To this end it was shown that intracerebral delivery of lentiviral vectors driving the expression of KLK6 reduced the accumulation of intracellular α-synuclein and ameliorated the neuropathology in a transgenic mouse model of dementia with Lewy bodies that overexpressed wt α-synuclein, although the mechanism underlying the beneficial effect of KLK6 was not investigated [21]. Later, a lentiviral vector designed to express a fusion of KLK6 protein with ApoB was peripherally administered to a mouse model of MSA. It was demonstrated that the fused KLK6-ApoB could cross the blood-brain barrier resulting in lower levels of α-synuclein in oligodendrocytes and astrocytes as well as behavior improvement [42]. Although *in vitro* studies have implicated KLK6 in PAR-mediated inflammation [43], no signs of neuroinflammation were observed following KLK6 delivery to the mice brain [21, 42]. Taken together, these two studies indicated that KLK6 could represent a novel therapeutic protein for PD and other synucleinopathies.

## MATERIALS AND METHODS

### Materials

All chemicals were obtained from Sigma-Aldrich. The following antibodies were used: anti-KLK6 IgY (chicken antibody): its production and characterization has been described previously [44]; anti-TIMP2 (rabbit polyclonal, Abcam); anti-MMP2 (D8N9Y) and MMP9 (G657) (rabbit polyclonals, Cell Signaling); anti-α-synuclein, Syn-1 (mouse monoclonal, BD Transduction Laboratories); anti-α-synuclein, (C-20)-R (rabbit polyclonal, Santa Cruz); anti-α-synuclein, 4B12 (mouse monoclonal, Gene Tex); anti-BSA (mouse monoclonal, AbD Serotec); anti-β-actin (mouse monoclonal, Sigma-Aldrich); anti-proMMP2 antibody was a gift from Dr. Pieter Koolwijk (VU University Medical Center, Amsterdam, The Netherlands) and its specificity was validated in breast cancer cell supernatants that expressed proMMP2. Secondary antibodies (HRP-conjugated) were obtained from Jackson ImmunoResearch Laboratories or Merck. Recombinant KLK6 R80Q (active) engineered for resistance to self-inactivation and KLK6 S197A that is inactive due to a point mutation at the active site Ser, were produced in our laboratory as described [29]. Recombinant forms of α-synuclein were kindly provided by El-Agnaf M.A. Omar (Neurological Disorders Center, Qatar Biomedical Research Institute - QBRI).

### Cell culture

The generation of stable SH-SY5Y cell lines inducibly overexpressing human wild-type α-synuclein has been described previously [25]. Cells were cultured in RPMI 1640 supplemented with 10% FBS and 100 μg/ml penicillin/streptomycin in the presence of 200 μg/ml geneticin and 50 μg/ml hygromycin B. The α-synuclein expression was switched off by addition of 1 μg/ml doxycycline (dox+).

### Animals

Klk6 knockout (*Klk6^–/–^)* mice on C57BL/6 background were generated in collaboration with Prof. Nagy and Dr. Michael (Mount Sinai Hospital, Toronto, Canada). These mice are viable, fertile and have no prominent phenotypic abnormalities. Wild-type littermates were used as control for our experiments. Animals were housed in the animal facility of the Biomedical Research Foundation of the Academy of Athens (BRFAA) in a room with a controlled light-dark cycle (12 hours light-12 hours dark) and free access to food and water. The competent Regional Veterinary authority approved the experimental protocol in accordance to Greek legislation (Presidential Decree 56/2013, in compliance with the European Directive 2010/63).

### Primary cultures of cortical neurons

Primary cortical neurons were prepared from mice (embryonic day 16) as previously described [45]. Briefly, dissociated cells were plated onto poly-D-lysine-coated 6-well dishes at a density of ~1,6 × 10^6^ cells per cm^2^ and maintained in Neurobasal medium (Gibco, Invitrogen), containing B27 serum-free supplements (Invitrogen), 0.5 mM L-glutamine, and penicillin/streptomycin.

### APMA activation

4-aminophenylmercury acetate was dissolved in DMSO at 20 mM. APMA was added to proMMP2 purified from SH-SY5Y supernatants or to SH-SY5Y secretome at 1 mM final concentration and samples were incubated at 37^°^C for the indicated times.

### Zymography

Cell supernatants were concentrated by ultrafiltration using Amicon filters with a cutoff of 10 kDa (Merck-Millipore), mixed with Laemmli buffer without mercaptoethanol for 15 min at 37°C, and resolved by SDS-PAGE in 12% acrylamide gels containing 0.1% gelatin. Gels were washed twice with 50 mM Tris-HCl pH 7.5, 5 mM CaCl_2_, 2.5% Triton X-100 for 15 min, 15 min with 50 mM Tris-HCl, pH 7.5, 5 mM CaCl_2_, 0.1% Triton X-100, then, incubated in the latter buffer for 24 hours at 37^°^C and, finally, stained with Coomassie G-250 or R-250.

### Proteolysis of recombinant α-synuclein forms

Recombinant α-synuclein forms (monomeric and fibrilar) were incubated with the active (R80Q) form of KLK6 at 37^°^C at the indicated concentrations and for various time intervals. Upon completion, reaction mixtures were resolved by SDS-PAGE and immunoblotted for α-synuclein.

### Purification of proMMP2

Endogenous proMMP2 was purified from SH-SY5Y supernatants by a two-step affinity chromatography. Briefly, supernatants were concentrated by ultrafiltration and dialyzed against 50 mM Tris.HCl, pH 7.5, 0.5 M NaCl, 0.05% Triton X-100. Samples were loaded on 3 ml Gelatin-Sepharose 4B column and eluted in 50 mM Tris.HCl, pH 7.5, 1 M NaCl, 5% DMSO. Fractions containing MMPs were identified by gelatin zymography. Concanavalin A-Sepharose 4B was used in the second step. Samples were dialyzed against 50 mM Tris.HCl, pH 7.5, 0.15 M NaCl, 10 mM CaCl_2_, 0.05% Triton X-100 and loaded on 3 ml column. The non-binding fraction (proMMP2) was collected and dialyzed against 50 mM Tris.HCl, pH 7.5, 0.15 M NaCl, 10 mM CaCl_2_.

### Addition of exogenous α-synuclein forms to primary neuronal cultures and sequential protein extraction of primary neurons

Primary cortical neurons were plated in 6-well plates and treated with human monomeric α-synuclein, specific human strains of fibrilar α-synuclein (fibrils and ribbons) and mouse fibrilar α-synuclein. After 3 or 4 days of treatment, neurons were mildly rinsed with trypsin to wash out the excess of proteins not attached to the cells and sequential extraction of proteins was performed. Briefly, neuronal pellets were resuspended in ice-cold 1% TX-100 containing protease and phosphatase inhibitors and were sonicated 5 times (0.5 sec pulse at 20% power). Lysates were incubated for 30 min on ice and were cleared by centrifugation at 10,000 × g for 30 min at 4^°^C. Resulting supernatants comprise TX-100-soluble fractions, containing cytosolic, soluble proteins. The remaining pellets were washed twice and then were resuspended in 1% sarcosyl lysis buffer, containing protease and phosphatase inhibitors. Lysates were sonicated 5 times (2 sec pulse at 50% power), following a 30 min incubation on ice and then were centrifuged at 13000 × g for 30 min at 4^°^C. The resulting supernatants comprise sarcosyl extracts, containing TX-100 insoluble and membrane-associated proteins. Protein content was estimated using the Bradford method (Bio-Rad, Hercules, CA, USA).

### SDS-PAGE and immunoblotting

Denaturing gel electrophoresis was performed as indicated either on 13% SDS-PAGE Tris-glycine gels or on 4–12% NuPAGE Bis-Tris gels (Life Technologies). Proteins were subsequently transferred onto nitrocellulose membranes (0.45 mm pore size) (Whatman) and analyzed by immunoblotting. All immunoblots represent one of at least three independent experiments.

### Recombinant adenovirus construction

The cDNA encoding the full-length preproKLK6 with mutations R80Q (active) or S197A (inactive) were cloned into pcDNA3.1(+). The inserts were subcloned into pGD-Entry with the restriction enzymes *Xma*I and *Eco*RI and the genes were transferred into the pAd-PL-DEST vector using the Gateway system (Invitrogen). The vector was linearized with *Pac*I, purified by phenol/chloroform extraction, and transfected into packaging HEK293 cells by calcium phosphate precipitation. Viral particles were amplified from plaque isolates in order to guarantee homogeneity of the production. Final viral stocks were collected after lysing the cells by freezing-thawing and, subsequently, were purified and concentrated by double discontinuous and continuous cesium chloride (CsCl) gradients. The titers of the purified viral vector stocks were determined using the Adeno-X Rapid (Clontech). The following titers were obtained, expressed as viral particles/μl: 1.1×10^8^ for preproKLK6 R80Q and 7.9 × 10^7^ for preproKLK6 S197A.

### Terminal amine isotopic labeling of substrates (TAILS)

For TAILS, 1 mg of CM was treated with 50 nM active or inactive KLK6 for 24 hours. TAILS was then performed as described previously [46].

## SUPPLEMENTARY MATERIALS FIGURES AND TABLES









## Abbreviations

ADAMTS19: a disintegrin and metalloproteinase with thrombospondin motif 19
APMA: 4-aminophenylmercury acetate
CM: conditioned media
CSF: cerebrospinal fluid
KLK6: kallikrein-related peptidase 6
LB: Lewy body
MSA: multiple system atrophy
proMMP2: (pro matrix metalloproteinase 2)
PD: Parkinson disease
PFF: pre-formed fibrils
TAILS: terminal amine isotopic labeling of substrates
