# Supplementary material for: KLK6 proteolysis is implicated in the turnover and uptake of extracellular alpha-synuclein species
Source: Oncotarget. 2016 Nov 10;8(9):14502–15. doi: 10.18632/oncotarget.13264 (PMC5362421; doi:10.18632/oncotarget.13264)
Supplement: Supplementary file 1 [file oncotarget-08-14502-s001.pdf]

KLK6 proteolysis is implicated in the turnover and uptake of extracellular alpha-synuclein species

Supplementary Materials

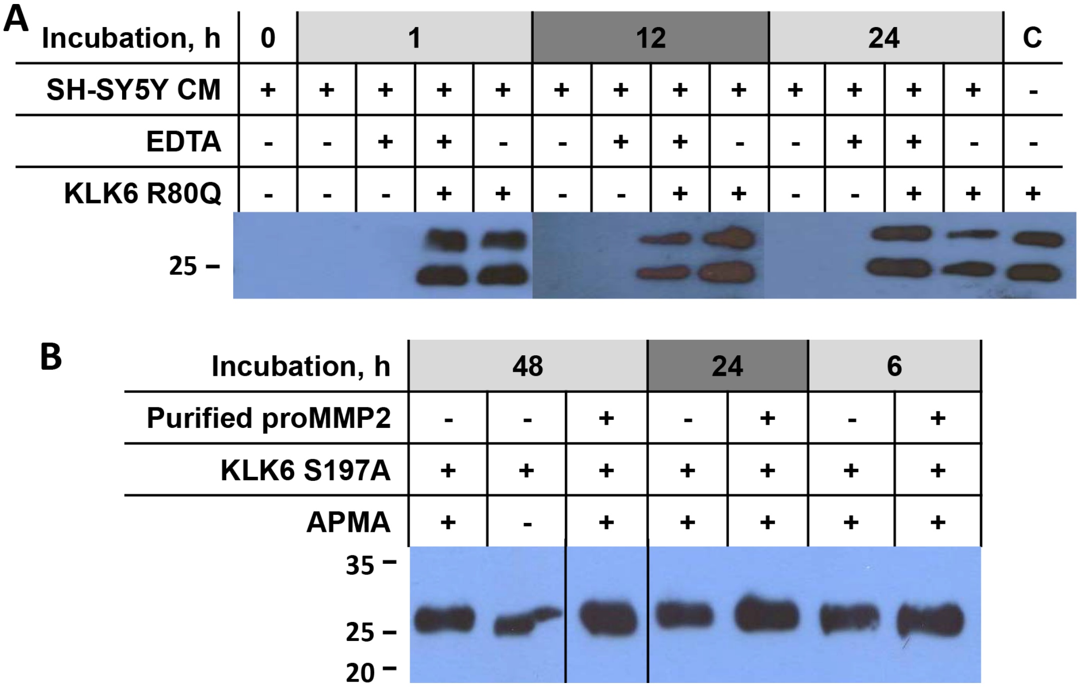

Supplementary Figure S1: Stability of KLK6. (**A**) KLK6 is active in the presence of CM from SH-SY5Y cells in the presence or absence of EDTA. (**B**) KLK6 is not degraded by MMP2. KLK6 S197A was used in order to avoid self-degradation upon extended incubation period (48 hours).

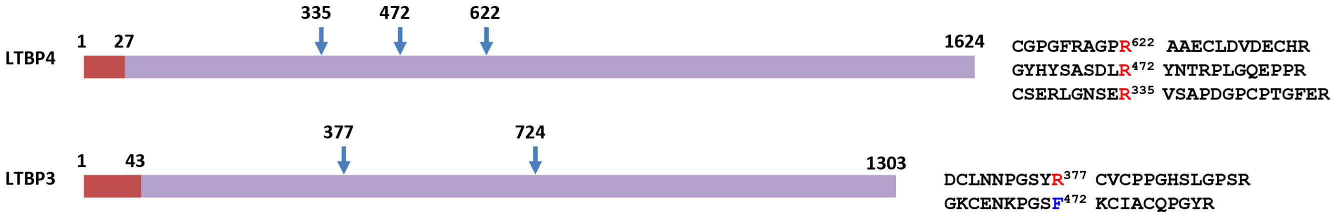

Supplementary Figure S2: Cleavage of latent TGF-β binding proteins by KLK6 as identified by TAILS.

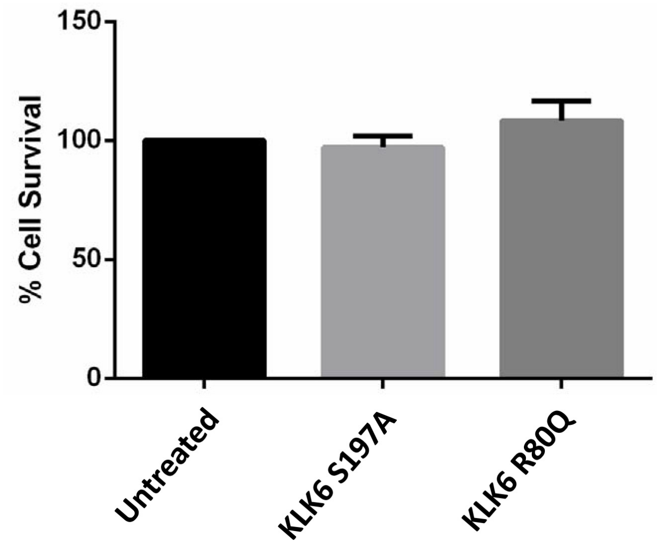

Supplementary Figure S3: Adenoviral expression of active KLK6 R80Q or inactive KLK6 S197A does not affect cell viability.

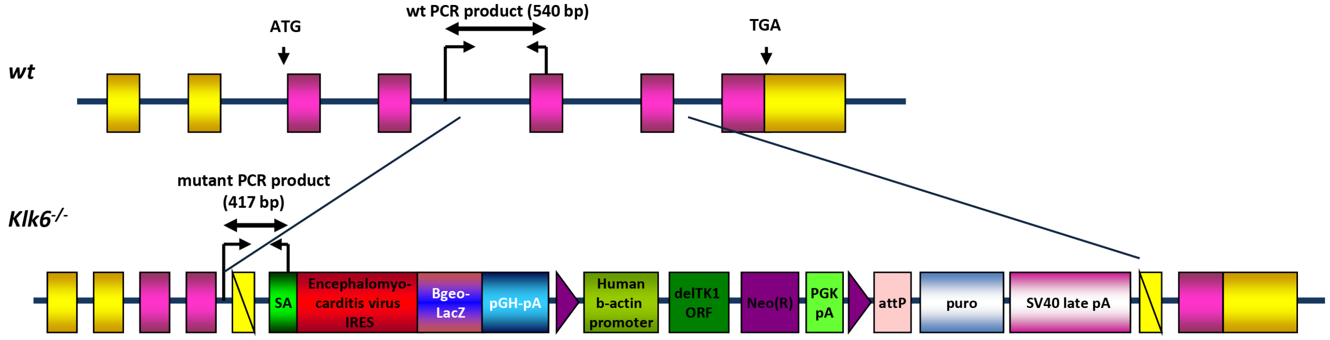

Supplementary Figure S4: Schematic diagram of the generation of *Klk6*$^{-/-}$ mice.

Supplementary Table S1: Results from the first TAILS analysis. See Supplementary_Table_S1

Supplementary Table S2: Results from the second TAILS analysis. See Supplementary_Table_S2

Supplementary Table S3: Cleavage sites identified by both TAILS with Fc > 2.
See Supplementary_Table_S3
